# Supplementary material for: Sub-4 nanometer porous membrane enables highly efficient electrodialytic fractionation of dyes and inorganic salts
Source: Nat Commun. 2025 Apr 17;16:3671. doi: 10.1038/s41467-025-58873-5 (PMC12006429; doi:10.1038/s41467-025-58873-5)
Supplement: Supplementary file 1 — Supplementary Information [file 41467_2025_58873_MOESM1_ESM.pdf]

Supplementary Materials for

Sub-4 nanometer porous membrane enables highly efficient electrodialytic fractionation of dyes and inorganic salts

Jiuyang Lin[1,2,3], Zijian Yu[3], Tianci Chen[2], Junming Huang[3], Lianxin Chen[3], Jiangjing Li[3], Xuewei Li[2], Xiaolei Huang[2], Jianquan Luo[4], Elisa Yun Mei Ang[5], William Toh[6], Peng Cheng Wang[5], Teng Yong Ng[6], Dong Han Seo[7], Shuaifei Zhao[8], Kuo Zhong[9]*, Ming Xie[10]*, Wenyuan Ye[1]*, Bart Van der Bruggen[11,12], Yinhua Wan[2]*

* Corresponding authors: kuo.zhong@hkait.cn (K. Zhong); mx406@bath.ac.uk (M. Xie); ye0508@126.com (W. Ye); yhwan@gia.cas.cn (Y. Wan)

[1] Jiangxi University of Science and Technology, Ganzhou 341000, China

[2] Key Laboratory of Rare Earths, Ganjiang Innovation Academy, Chinese Academy of Sciences, Ganzhou 341119, China

[3] School of Environment and Safety Engineering, Fuzhou University, Fuzhou 350116, China

[4] State Key Laboratory of Biopharmaceutical Preparation and Delivery, Institute of Process Engineering, Chinese Academy of Sciences, Beijing 100190, China

[5] Engineering Cluster, Singapore Institute of Technology, Singapore138683, Singapore

[6] School of Mechanical and Aerospace Engineering, Nanyang Technological University, Singapore 639798, Singapore

[7] Institute of Energy Materials & Devices, Korea Institute of Energy Technology (KENTECH), Naju, Republic of Korea

[8] Institute for Frontier Materials, Deakin University, Geelong VIC 3216, Victoria, Australia

[9] HuiKang Advanced Institute of Technology, Shenyang 110179, China

[10] Department of Chemical Engineering, University of Bath, Bath BA2 7AY, United Kingdom

[11] Department of Chemical Engineering, Process Engineering for Sustainable Systems (ProcESS), KU Leuven, B-3001 Leuven, Belgium

[12] Faculty of Engineering and the Built Environment, Department of Chemical, Metallurgical and Materials Engineering, Tshwane University of Technology, Pretoria 0001, South Africa

This PDF file includes:

1. Supplementary Methods

1.1 Membrane characterization

1.1.1 Specific areal resistance of the sub-4 nanometer porous membrane

1.1.2 Pore size and molecular weight cutoff of the sub-4 nanometer porous membrane

1.2 Molecular dynamics simulation

2. Supplementary Figures

2.1 Chemical composition of the tested membrane

2.2 Pressure-driven filtration performance in pure NaCl solutions with different salinities

2.3 Pressure-driven filtration performance in pure dye solutions

2.4 Dye aggregation behavior of reactive black 5 by molecular dynamics simulation

2.5 Dye rejection during pressure-driven filtration of reactive black 5 solutions by molecular dynamics simulation

2.6 NaCl/dye selectivity during pressure-driven filtration of dye/NaCl mixture solutions

2.7 Ion drift velocity in the electro-driven filtration of pure NaCl solution calculated by molecular dynamic simulation

2.8 Electro-driven filtration performance of the sub-4 nanometer porous membrane in pure NaCl solutions with different salinities

2.9 Permselectivity between NaCl and reactive black 5 during electro-driven filtration of dye/NaCl mixture solution using sub-4 nanometer porous membrane

2.10 Specific areal electric resistance of sub-4 nanometer porous membrane before and after fouling

2.11 Electro-driven filtration performance in the dye/NaCl mixture solution using commercial anion exchange membrane

2.12 Schematic of the pressure-driven constant-volume diafiltration process

2.13 Electro-driven filtration setup and sub-4 nanometer porous membrane coupon as anion conducting membrane

2.14 Schematic of four compartment device for measurement of specific areal resistance of the anion conductive membranes

2.15 Performance of the pressure-driven constant-volume diafiltration and electro-driven filtration process for fractionation of dye and NaCl

1. Supplementary methods

1.1 Membrane characterization

1.1.1 Specific areal resistance of the sub-4 nanometer porous membrane

An electrical resistance measurement device (ChemJoy Polymer Material Co., Ltd., China) was employed to determine the specific areal resistance of anion conducting membranes (including sub-4 nanometer porous membrane and commercial anion exchange membrane) before and after the electrodialytic filtration in the dye/NaCl mixture solution (**Supplementary Fig. 14**). The commercial cation exchange membrane (TWEDC1S70, Shandong Tianwei Membrane Technology Co., Ltd., China) was employed as an auxiliary membrane to be inserted between the cathode/anode and the tested anion conducting membrane (i.e., sub-4 nanometer porous membrane or commercial anion exchange membrane with effective area of 7.065 cm^2) for effective isolation between NaCl testing solution and Na_2SO_4 electrolyte solution. Specific areal resistance (R_s) of the anion conducting membrane was determined at a fixed current of 0.1 A through Eq. S1[1]:

$$R_s = \frac{V_M - V_0}{I} \cdot A \quad \text{(S1)}$$

where V_M is the trans-membrane voltage for the tested anion conducting membrane; V_0 is the blank voltage with no anion conducting membrane; I is the applied current intensity during the measurement; A is effective area of the anion conducting membrane.

1.1.2 Pore size and molecular weight cutoff of the sub-4 nanometer porous membrane

Filtration of 0.2 g L^{-1} poly(ethylene glycol) solutions with molecular weights (MW) of 600, 1000, 1500, 2000, and 4000 g mol^{-1} was conducted at 4 bar to measure the pore size of the anion conducting membrane. Generally, the relationship between the Stokes diameter (d_s) of the poly(ethylene glycol) polymers and their MW can be described by Eq. S2:

$$d_s = 33.46 \times 10^{-12} \times MW^{0.557} \quad \text{(S2)}$$

After the separation of the poly(ethylene glycol) solutions at 4 bar, the concentration of the poly(ethylene glycol) polymers in the feed (C_f) and permeate (C_p) was determined by a total organic carbon analyzer (TOC-L CpH/CPN, Shimadzu, Japan). The rejection coefficient (R_p) of the poly(ethylene glycol) polymers for the anion conducting membrane can be calculated by Eq. S3:

$$R_p = 1 - \frac{C_p}{C_f} \quad \text{(S3)}$$

In addition, a log-normal probability density function between the Stokes diameter (d_p) of the poly(ethylene glycol) polymers and their rejection coefficients (R) was applied to determine the pore size distribution by Eq. S4[2]:

$$\frac{\mathrm{d}R\left(d_{\mathrm{p}}\right)}{\mathrm{d}\left(d_{\mathrm{p}}\right)}=\frac{1}{d_{\mathrm{p}}\ln\sigma_{\mathrm{p}}\sqrt{2\pi}}\exp\left[-\frac{\left(\ln d_{\mathrm{p}}-\ln\mu_{\mathrm{p}}\right)^{2}}{2\left(\ln\sigma_{\mathrm{p}}\right)^{2}}\right] \tag{S4}$$

where μ_{p} is the mean effective pore size of the tested anion conducting membrane, which is defined at the rejection of 50.00%. σ_{p} is the geometric standard deviation for pore size of the membrane, which is defined as the ratio between the pore size of the membrane at the rejection of 84.13% and 50.00%.

Moreover, the molecular weight cutoff of the tested membrane was back-calculated based on the solute Stokes diameter at the rejection of 90% through Eq. S2.

1.2 Molecular dynamics simulation

Molecular dynamics simulation was conducted to unravel the ion transfer of the sub-4 nanometer porous membrane in both pressure-driven and electro-driven filtration process. In this work, the Large-scale Atomic/Molecular Massively Parallel Simulator (LAMMPS) was used for all molecular dynamics simulations[3]. The visualizations were performed using the Open Visualization tool (Ovito). The simulation domain is setup as seen in **Figure 2C** for the pressure-driven filtration process, and **Figure 5C** for the electro-driven filtration process.

A 90-monomer polyethersulfone-based chain was built using PubChem, Avogadro[4] and Moltemplate[5]. The 90-monomer polyethersulfone-based chain is used to form a polyethersulfone-based membrane of size 5 nm by 5 nm by 1.2 nm with a 2.49 nm diameter hole. The general amber force field (GAFF) was used to model the intra and inter molecular interactions of the nanoporous polyethersulfone-based membrane[6]. Similarly, the molecular structure of reactive black 5 dye was obtained from PubChem and Moltemplate was used to generate the required GAFF force field to model the inter and intra molecular interactions. A total of 4 molecules of reactive black 5 dye were added to the feed section of the domain.

In the pressure-driven filtration process, the feed section of the domain in front of the membrane was initially set to a volume of 12 nm by 5 nm by 5 nm, while the permeate section after the membrane was set to 0.8 nm by 5 nm by 5 nm. The simulation domain was held in place with two graphene pistons along the longitudinal axis. Periodic boundary conditions were employed in the domain. However, sufficient distance between the two graphene pistons was maintained in the domain to prevent interactions of the feed and permeate across the boundaries. On the other hand, in the electro-driven filtration process, a domain with a length of 17 nm was constructed. Periodic boundary conditions were employed for all three axes. Water molecules were modelled using the TIP3P model with a long-range coulombic solver[7]. The particle-particle particle-mesh solver (PPPM) long-range coulombic solver was employed for this study. The monovalent salt ion interactions were modelled using with Lennard-Jones interactions with coulombic potential, with their parameters obtained from Joung et al[8]. The intermolecular interactions are modelled using a mixing rule for the Lennard-Jones interactions. The global cutoff distance for Lennard-Jones and Coulombic forces were

specified to be 9 Å and 10 Å, respectively. In the pressure-driven filtration process, two separate sets of simulations were run. In the first set, the feed domain only consists of 4 molecules of reactive black 5 dye and pure water molecules. Simultaneously, in the second set, the feed domain consisted of 4 molecules of reactive black 5 dye in a 14.6 g L^{-1} NaCl solution. These 4 molecules of reactive black 5 dye were initially placed separated, but close to the membrane. In these two sets, the nanoporous polyethersulfone-based membrane was fixed in the domain. A canonical NVT ensemble was used in this simulation, where the moles, volume and temperature were kept constant throughout. Temperature was kept constant at 300K throughout the simulation. A time step of 1 femto-second (fs) is adopted for this simulation. An equilibration where the reactive black 5 molecules and graphene pistons were fixed. Water/salt mixture was allowed to relax in the domain, which was carried out for 0.5 ns. Thereafter, the reactive black 5 molecules were unfrozen and allowed to equilibrate for a next 0.5 ns. In the extra 4 ns, the graphene pistons were unfrozen and allowed to move in the longitudinal dimension under zero pressure differential between the left and right piston. Finally, from 5 ns, the system was given a pressure differential of 26.7 MPa across the two graphene pistons. A larger pressure differential was required so that the interactions can be observed in a reasonable computational time. Such a technique has been applied and documented in several molecular dynamics studies involving permeable membrane[9, 10]. Results were collected from 13 ns to 21 ns (8 ns of permeation time).

In the electric-driven filtration process, 4 molecules of reactive black 5 dye in a 14.6 g L^{-1} NaCl solution were modelled. The nanoporous polyethersulfone-based membrane was fixed in the middle of the domain. A canonical NVT ensemble was used in this simulation with temperature thermostat at 300 K throughout the simulation. A time step of 1 femto-second is adopted for this simulation. An equilibration with the reactive black 5 dye molecules fixed were carried out for 1 ns. Thereafter, reactive black 5 molecules were unfrozen and the system equilibrated for 5 ns. From 6 ns to 16 ns (10 ns of production run), an electric field of 0.1 V $Å^{-1}$ was applied across the domain. Such a large electric field was applied to ensure that the interactions can be observed in a reasonable computational time, like the case in the pressure-driven filtration process.

2. Supplementary data and figures

2.1 Chemical composition of the tested membrane

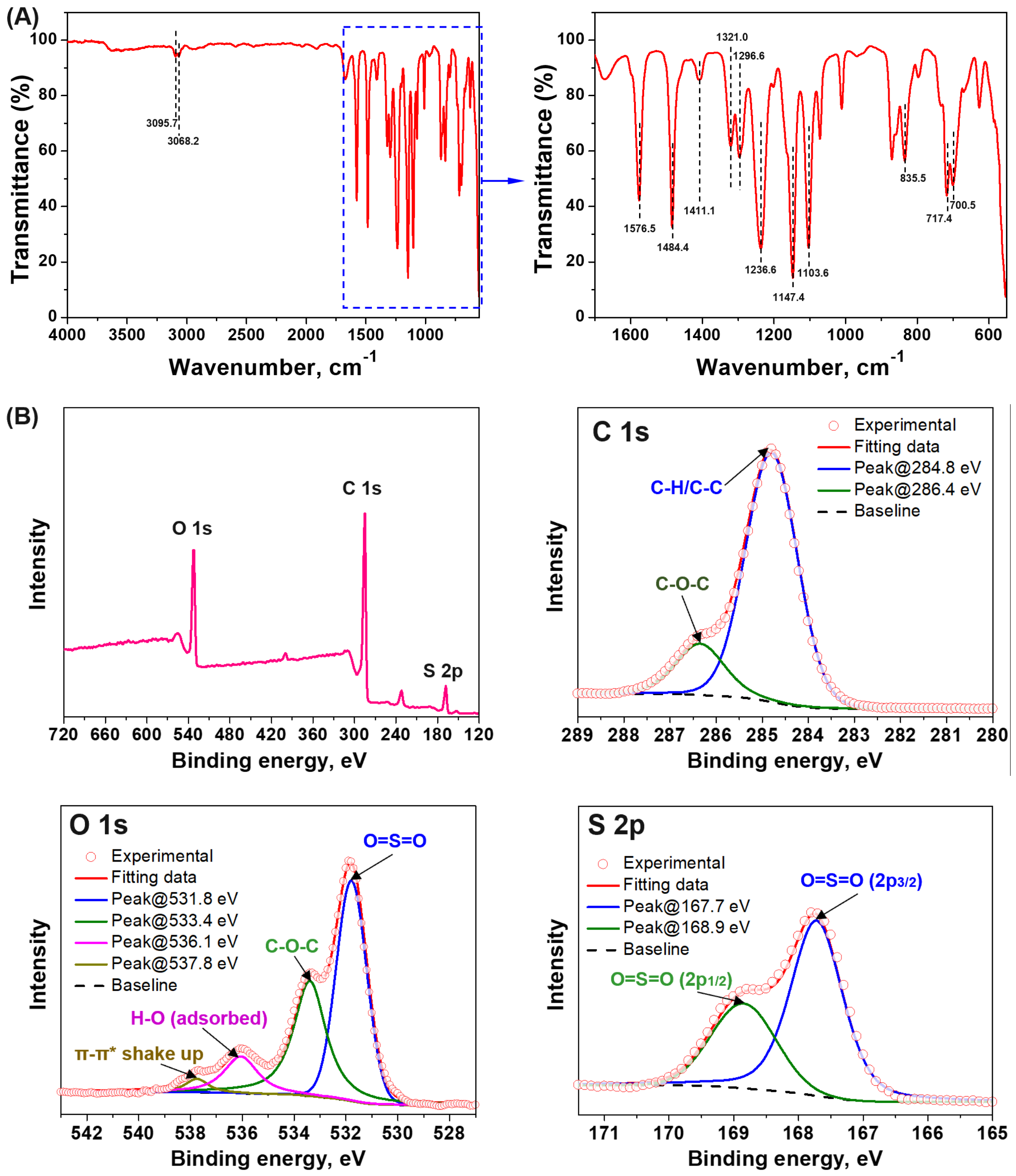

Supplementary Fig. 1. Chemical composition measurement of the tested membrane. (A) FTIR; **(B)** XPS wide scan and high-resolution scan spectra for C 1s, O 1s and S 2p.

The characteristic stretching vibrations of polyethersulfone (i.e., at the wavenumber of 1576.5, 1484.4, 1411.1, 1321.0, 1296.6, 1236.6, 1147.4, 1103.6 and 717.4 cm^{-1}) can be observed in the FTIR spectra of the tested membrane in this study. Specifically, three typical peaks at the wavenumbers of 1576.5, 1484.4 and 1411.1 cm^{-1} are assigned to aromatic ring groups (benzene rings)[11]; the intrinsic peaks at the wavenumbers of 1321.0/1296.6 cm^{-1} and 1147.4/1103.6 cm^{-1} are attributed to the asymmetric and

symmetric stretching vibrations of sulfone groups (Ar-SO_2-Ar group), respectively[11]; the wavenumber of 717.4 cm^{-1} represents the stretching vibrations of C-S-C group[12]; a strong peak at the wavenumber of 1236.6 cm^{-1} is associated to aromatic ether structure (Ar-O-Ar group)[11,12]; in addition, the wavenumbers of 3095.7, 3068.2, 835.5 and 700.5 cm^{-1} are due to the stretching vibration of C-H group[13].

2.2 Pressure-driven filtration performance in pure NaCl solutions with different salinities

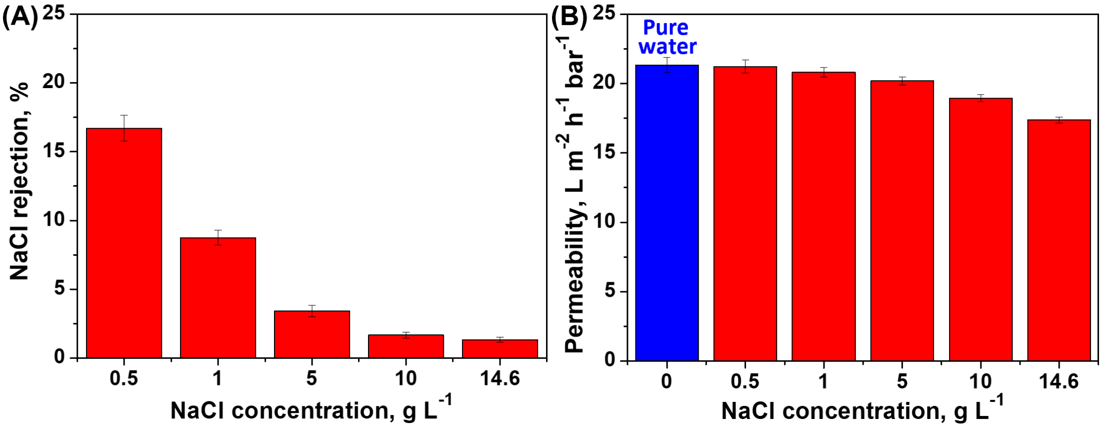

Supplementary Fig. 2. Pressure-driven filtration performance of the sub-4 nanometer porous membrane in pure NaCl solution with different salinities. (A) NaCl rejection; **(B)** Permeability (error bars in the figure represent standard deviation of three measurements).

2.3 Pressure-driven filtration performance in pure dye solutions

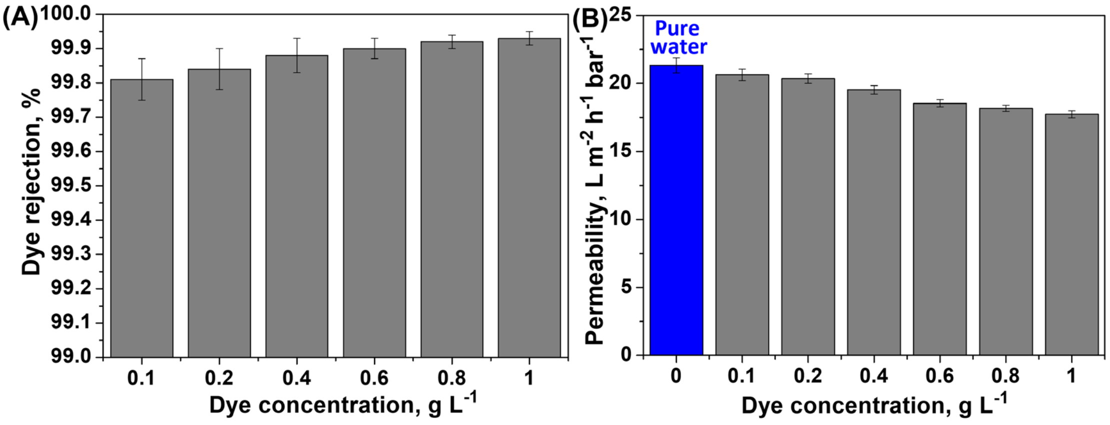

Supplementary Fig. 3. Pressure-driven filtration performance of the sub-4 nanometer porous membrane in pure reactive black 5 solutions with different concentrations. (A) Dye rejection; **(B)** Permeability (error bars in the figure represent standard deviation of three measurements).

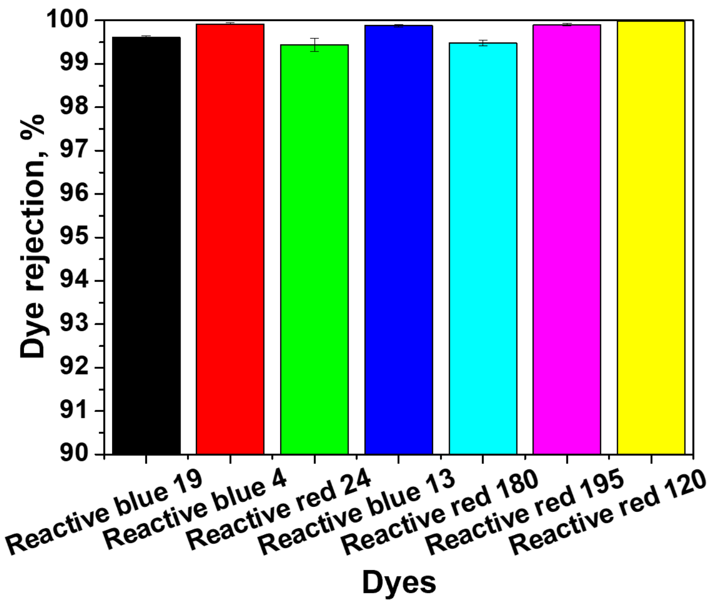

Supplementary Fig. 4. Pressure-driven separation performance of the sub-4 nanometer porous membrane in different reactive dye solutions (dye concentration of 1.0 g L^{-1}) (error bars in the figure represent standard deviation of three measurements).

2.4 Dye aggregation behavior of reactive black 5 by molecular dynamics simulation

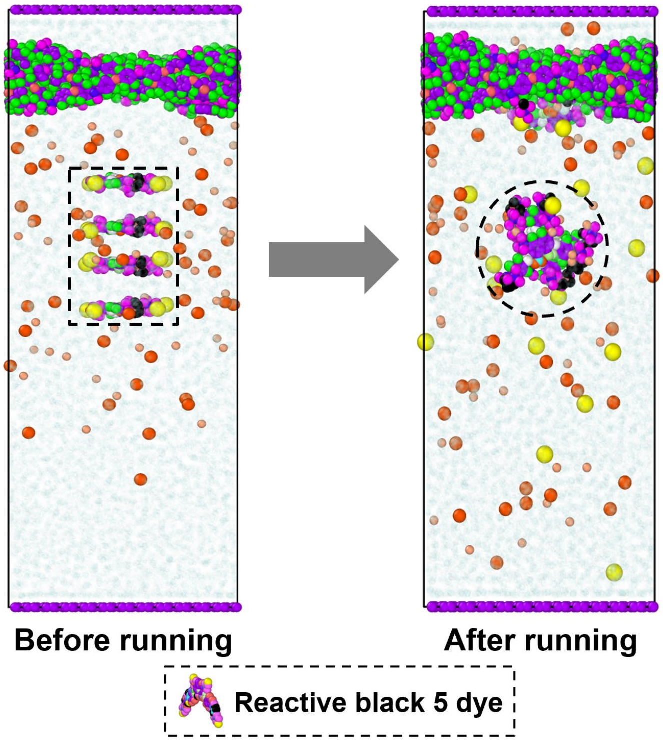

Supplementary Fig. 5. Aggregation of reactive black 5 molecules for dye cluster formation via molecular dynamics simulation

2.5 Dye rejection during pressure-driven filtration of reactive black 5 solutions by molecular dynamics simulation

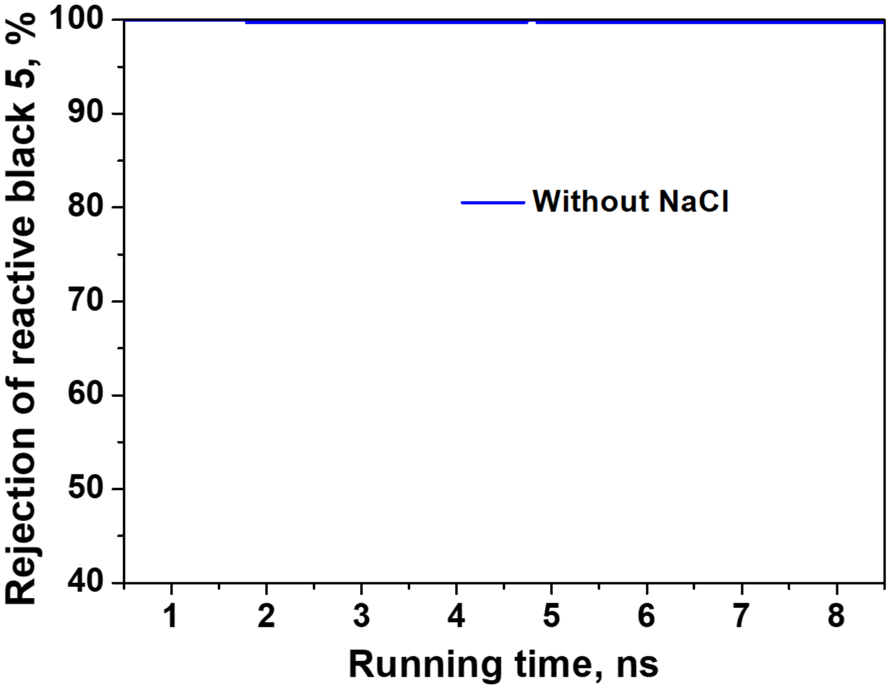

Supplementary Fig. 6. Rejection of reactive black 5 during the pressure-driven filtration of pure reactive black 5 solution by molecular dynamics simulation

2.6 NaCl/dye selectivity during pressure-driven filtration of dye/NaCl mixture solutions

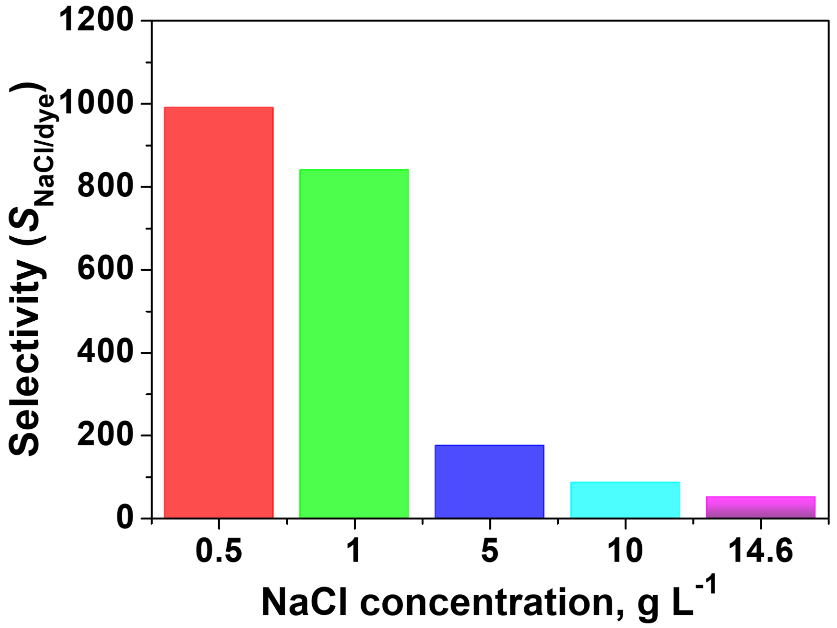

Supplementary Fig. 7. The selectivity between NaCl and reactive black 5 of the sub-4 nanometer porous membrane during the pressure-driven filtration of the dye/NaCl mixture solutions at different salinities

2.7 Ion drift velocity in the electro-driven filtration of pure NaCl solution calculated by molecular dynamic simulation

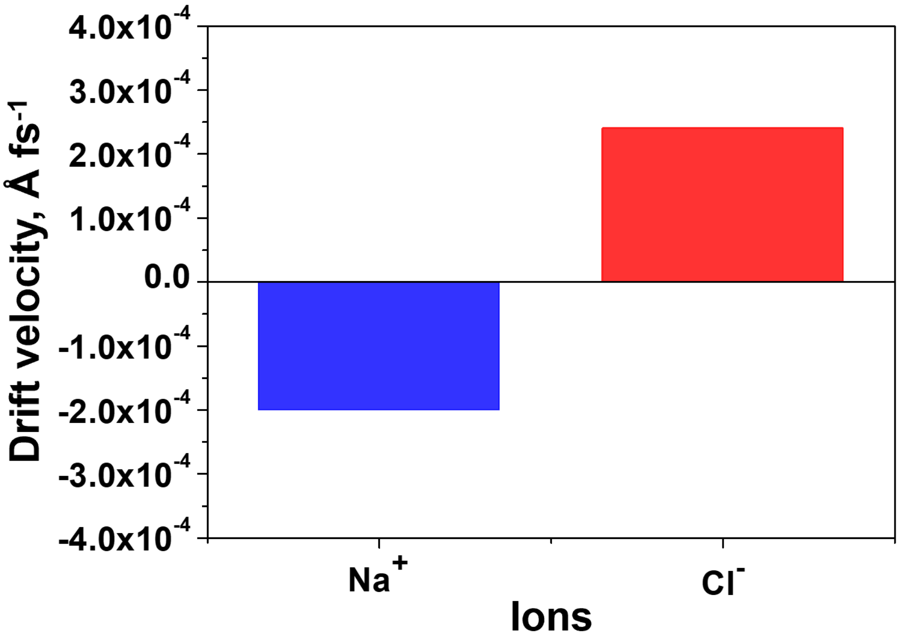

Supplementary Fig. 8. Drift velocity of Na^+ and Cl^- ions calculated by molecular dynamic simulation in the electro-driven filtration system equipped the sub-4 nanometer porous membrane as anion conducting membrane

2.8 Electro-driven filtration performance of the sub-4 nanometer porous membrane in pure NaCl solutions with different salinities

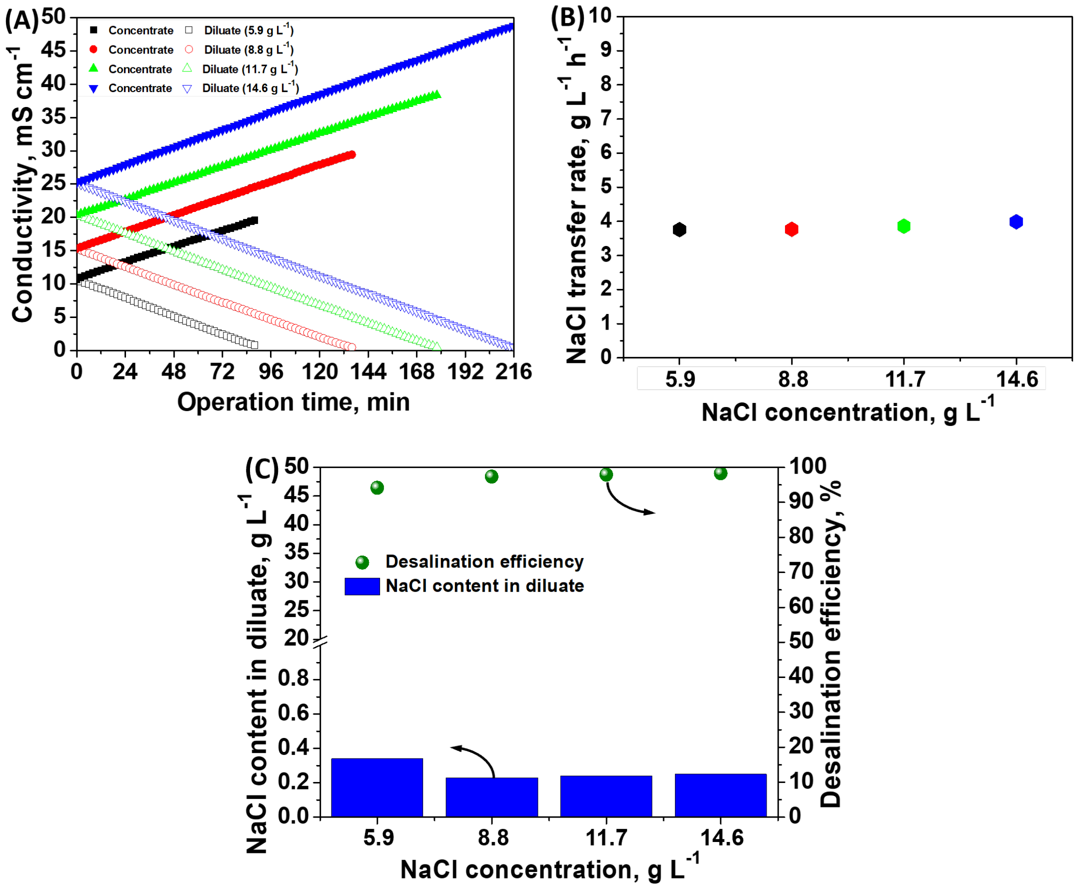

Supplementary Fig. 9. Electro-driven filtration performance of the sub-4 nanometer porous membrane as a role of anion conducting membrane in pure NaCl solutions with different salinities. (**A**) Evolution of conductivity in both concentrate and diluate solutions; (**B**) Electro-driven transfer rate of Cl^- ions through the sub-4 nanometer porous membrane; (**C**) NaCl concentration in the diluate and desalination efficiency.

2.9 Permselectivity between NaCl and reactive black 5 during electro-driven filtration of dye/NaCl mixture solution using sub-4 nanometer porous membrane

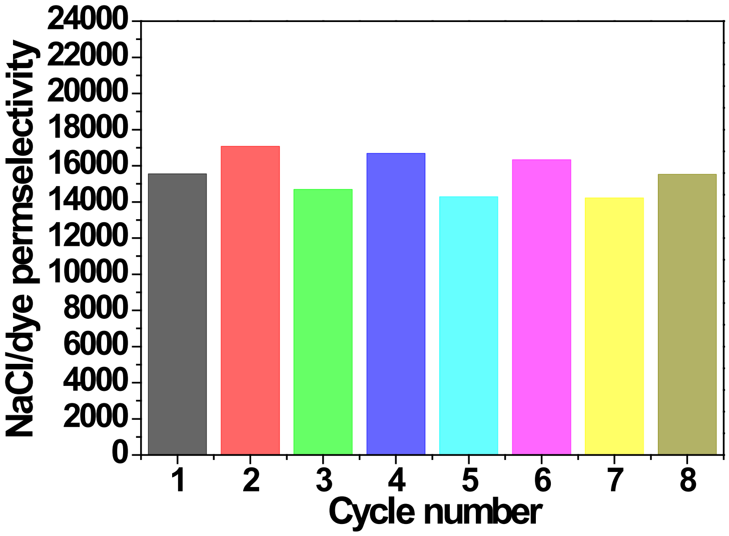

Supplementary Fig. 10. The permselectivity between NaCl and reactive black 5 dye for the sub-4 nanometer porous membrane as anion conducting membrane during an eight-cycle electrodialytic fractionation of reactive black 5 and NaCl in the reactive dye/NaCl mixture solution.

2.10 Specific areal electric resistance of sub-4 nanometer porous membrane before and after fouling

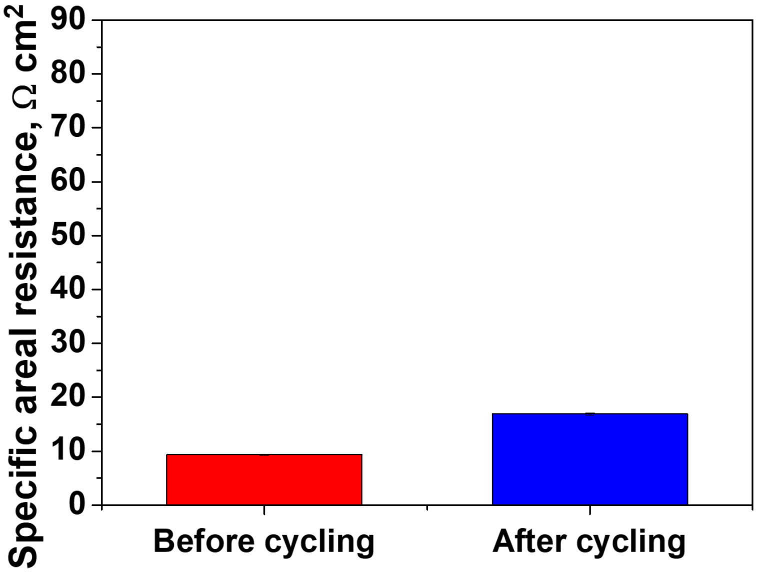

Supplementary Fig. 11. Specific areal electric resistance of sub-4 nanometer porous membrane before and after fouling during an eight-cycle electrodialytic filtration of the reactive dye/NaCl mixture solution (error bars in the figure represent standard deviation of three measurements).

2.11 Electro-driven filtration performance in the dye/NaCl mixture solution using commercial anion exchange membrane

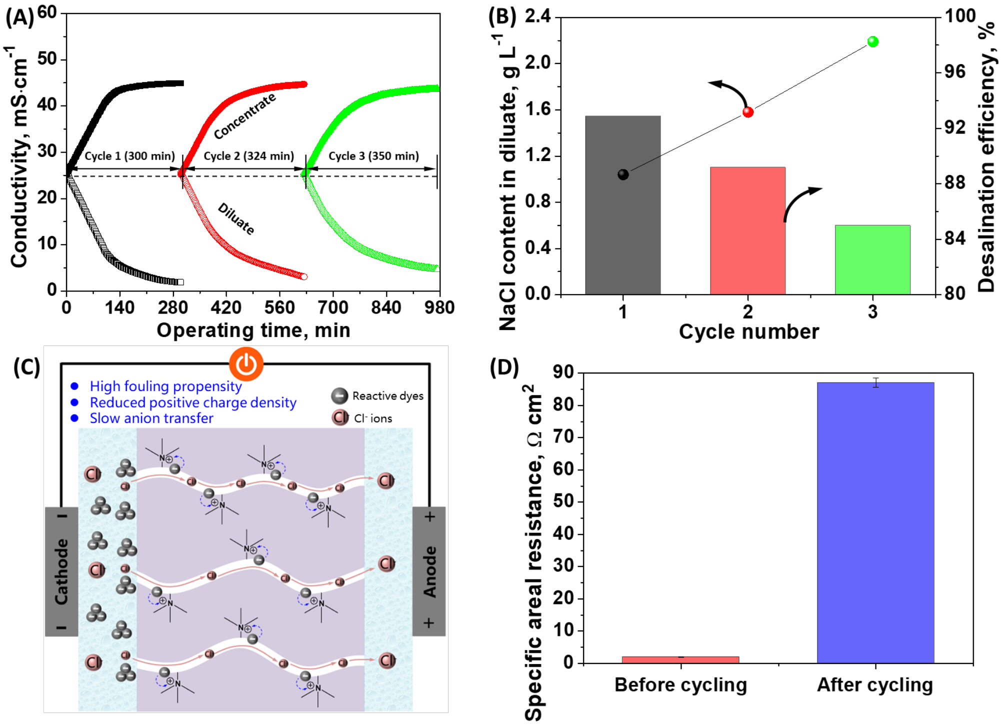

Supplementary Fig. 12. Three-cycle electrodialytic filtration using commercial anion exchange membrane for fractionation of dye and NaCl from the reactive black 5/NaCl mixture solution. (A) Evolution of conductivity in the concentrate and diluate solutions; **(B)** NaCl content in the diluate and desalination efficiency; **(C)** Illustration of anion (Cl^- and reactive black 5) transfer of commercial anion exchange membrane during electrodialytic fractionation of the reactive black 5/NaCl mixture solution; **(D)** Specific areal electric resistance of commercial anion exchange membrane before and after fouling (error bars in the figure represent standard deviation of three measurements).

2.12 Schematic of the pressure-driven constant-volume diafiltration process

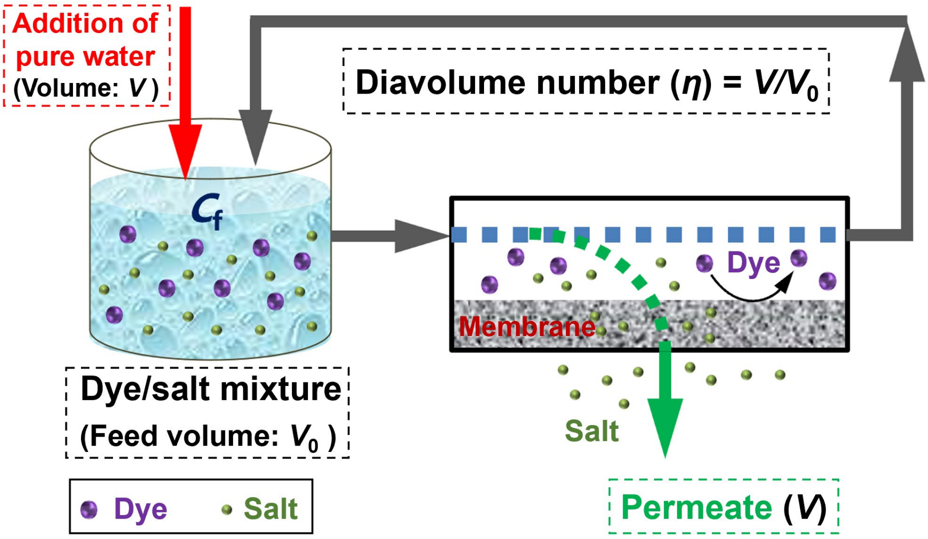

Supplementary Fig. 13. Schematic of the pressure-driven constant-volume diafiltration process using the sub-4 nanometer porous membrane in the reactive black 5/NaCl mixture solution for fractionation of dye/NaCl mixture.

2.13 Electro-driven filtration module and sub-4 nanometer porous membrane coupon as anion conducting membrane

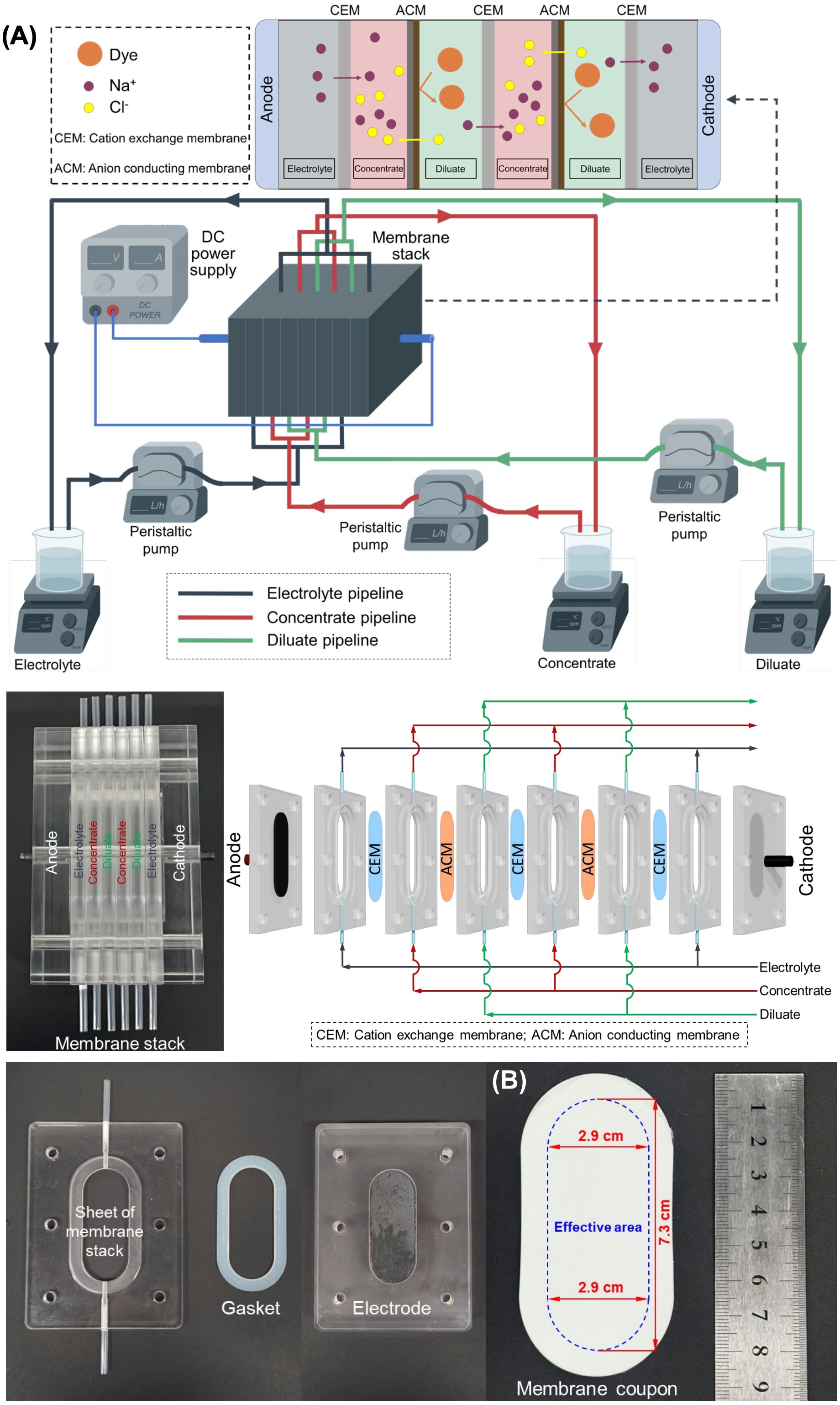

Supplementary Fig. 14. Electro-driven filtration system for fractionation of dye/salt mixture solution. (A) Schematic of electro-driven filtration setup and membrane stack; (B) Image of sub-4 nanometer porous membrane coupon as anion conducting membrane.

2.14 Schematic of four compartment device for measurement of specific areal resistance of the anion conductive membranes

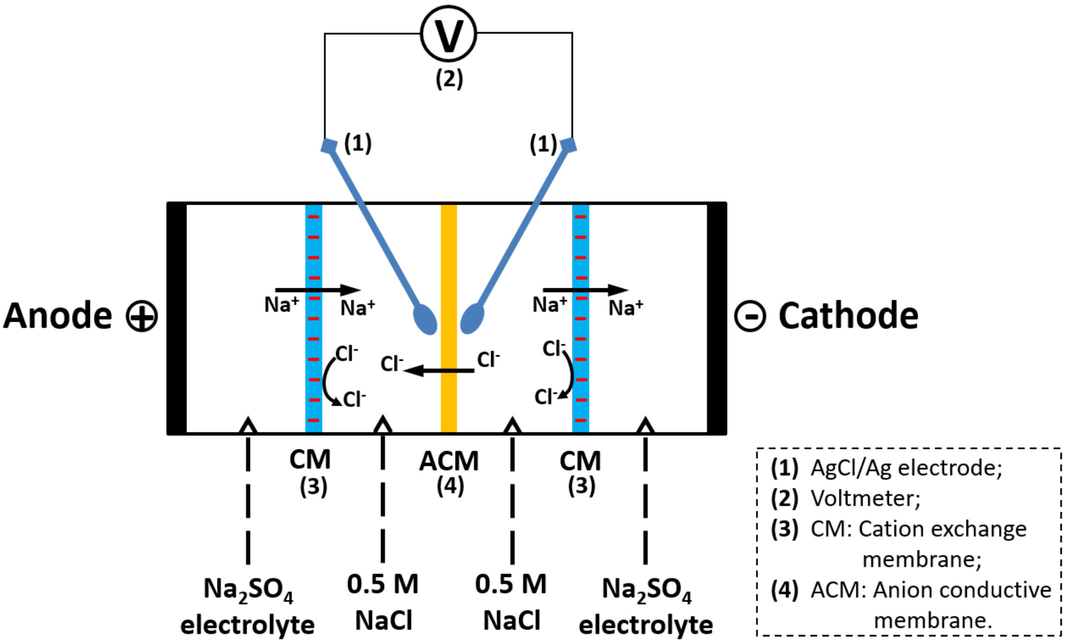

Supplementary Fig. 15. Schematic of four compartment device for measurement of specific areal resistance of the tested anion conducting membrane (CM: Cation exchange membrane; ACM: Anion conducting membrane).

2.15 Performance of the pressure-driven constant-volume diafiltration and electro-driven filtration process for fractionation of dye and NaCl

Supplementary Table 1. Performance of the pressure-driven diafiltration and electro-driven filtration process for fractionation of reactive black 5 dye and NaCl using the sub-4 nanometer porous membrane

Mode	NaCl content in feed, g L^{-1}	Desalination efficiency, %	Recovery of reactive dye, %
Pressure-driven diafiltration	0.28	98.07	98.29
Electro-driven filtration	0.27	98.15	99.66

Supplementary References

1. Zhao, Y. et al. A chemically assembled anion exchange membrane surface for monovalent anion selectivity and fouling reduction. *Journal of Materials Chemistry A* **7**, 6348-6356 (2019).
2. Ren, Y. et al. Tuning pore size and surface charge of poly(piperazinamide) nanofiltration membrane by enhanced chemical cleaning treatment. *Journal of Membrane Science* **643**, 120054 (2022).
3. Thompson, A.P. et al. LAMMPS - a flexible simulation tool for particle-based materials modeling at the atomic, meso, and continuum scales. *Computer Physics Communications* **271**, 108171 (2022).
4. Stukowski, A. Visualization and analysis of atomistic simulation data with OVITO-the Open Visualization Tool. *Modelling and Simulation in Materials Science and Engineering* **18**, 015012 (2010).
5. Hanwell, M.D. et al. Avogadro: An advanced semantic chemical editor, visualization, and analysis platform. *Journal of Cheminformatics* **4**, 17 (2012).
6. Wang, J., Wolf, R.M., Caldwell, J.W., Kollman, P.A. & Case, D.A. Development and testing of a general amber force field. *Journal of Computational Chemistry* **25**, 1157-1174 (2004).
7. Price, D.J. & Brooks, C.L.I. A modified TIP3P water potential for simulation with Ewald summation. *The Journal of Chemical Physics* **121**, 10096-10103 (2004).
8. Joung, I.S. & Cheatham, T.E.I. Determination of alkali and halide monovalent ion parameters for use in explicitly solvated biomolecular simulations. *The Journal of Physical Chemistry B* **112**, 9020-9041 (2008).
9. Toh, W., Ang, E.Y.M., Ng, T.Y., Lin, R. & Liu, Z. Antifouling bilayer graphene slit membrane for desalination of nanoplastic-infested seawater: A molecular dynamics simulation study. *ACS Applied Materials & Interfaces* **14**, 43965-43974 (2022).
10. Ang, E.Y.M. et al. Carbon nanotube arrays as multilayer transverse flow carbon nanotube membrane for efficient desalination. *Journal of Membrane Science* **581**, 383-392 (2019).
11. Zhou, Z. et al. Unveiling the susceptibility of functional groups of poly(ether sulfone)/polyvinylpyrrolidone membranes to NaOCl: A two-dimensional correlation spectroscopic study. *Environment Science & Technology* **51**, 14342-14351 (2017).
12. Sharma, K. et al. Positively charged nanofiltration membranes for enhancing magnesium separation from seawater. *Desalination* **568**, 117026 (2023).
13. Pihlajamäki, A., Väisänen, P. & Nyström, M. Characterization of clean and fouled polymeric ultrafiltration membranes by Fourier transform IR spectroscopy–attenuated total reflection. *Colloids and Surfaces A: Physicochemical and Engineering Aspects* **138**, 323-333 (1998).
