# Supplementary material for: Sub-4 nanometer porous membrane enables highly efficient electrodialytic fractionation of dyes and inorganic salts
Source: Nat Commun. 2025 Apr 17;16:3671. doi: 10.1038/s41467-025-58873-5 (PMC12006429; doi:10.1038/s41467-025-58873-5)
Supplement: Supplementary file 2 — Description of Additional Supplementary Files [file 41467_2025_58873_MOESM2_ESM.pdf]

Description of Additional Supplementary Files

File name: Supplementary Movie 1
Description: Aggregation of reactive black 5 dyes

File name: Supplementary Movie 2
Description: Retention of reactive black 5 by the nanoporous membrane during filtration of pure dye solution

File name: Supplementary Movie 3
Description: Retention of reactive black 5 by the nanoporous membrane during filtration of dye/NaCl mixture solution

File name: Supplementary Movie 4
Description: Transfer of Na^+ and Cl^- under an applied electric field of 0.1 V Å-1 during the electro-driven filtration of pure NaCl solution

File name: Supplementary Movie 5
Description: Transfer of Na^+, Cl^- and reactive black 5 under an applied electric field of 0.1 V Å-1 during the electro-driven filtration of dye/NaCl mixture solution
